# Risk of lead exposure from wild game consumption from cross-sectional studies in Madre de Dios, Peru

**DOI:** 10.1016/j.lana.2022.100266

**Published:** 2022-05-08

**Authors:** Axel J. Berky, Emily Robie, Susy Navio Chipa, Ernesto J. Ortiz, Emma J. Palmer, Nelson A. Rivera, Ana Maria Morales Avalos, Joel N. Meyer, Heileen Hsu-Kim, William K. Pan

**Affiliations:** a Nicholas School of the Environment, Duke University, Grainger Hall, 9 Circuit Drive, Box 90328, Durham, NC 27708, USA; b Duke Global Health Institute, Duke University, NC 27080, USA; c Dirección Regional de Salud, Madre de Dios, Peru; d Duke Global Health Innovations Centre, Duke University, NC 27080, USA; e Civil Architectural and Environmental Engineering, University of Texas at Austin, TX 78712, USA; f Department of Civil and Environmental Engineering, Pratt School of Engineering, Duke University, Durham, NC 27708, USA; g Dirección Ejecutiva de Medicina Alternativa y Complementaria, Instituto Nacional de Salud, Lima, Peru

**Keywords:** Lead exposure, Wild game, Hunting, Dual exposures, Madre de Dios, Lead ammunition, Peru

## Abstract

**Background:**

Studies have shown elevated blood lead levels (BLL) in residents of remote communities in the Amazon, yet sources of lead exposure are not fully understood, such as lead ammunition consumed in wild game.

**Methods:**

Data was collected during two cross-sectional studies that enrolled 307 individuals in 26 communities. Regression models with community random effects were used to evaluate risk factors for BLLs, including diet, water source, smoking, sex, age, and indigenous status. The All-Ages Lead Model (AALM) from the Environmental Protection Agency (EPA) was used to estimate background and dose from wild game consumption.

**Findings:**

Indigenous status and wild game consumption were associated with increased BLLs. Indigenous participants had 2.52 μg/dL (95% CI: 1.95−3.24) higher BLLs compared to non-indigenous. Eating wild game was associated with a 1.41 μg/dL (95% CI: 1.20−1.70) increase in BLLs. Two or more portions per serving were associated with increased BLLs of 1.66 μg/dL (95% CI: 1.10−2.57), compared to smaller servings. Using the AALM, we estimate background lead exposures to be 20 μg/day with consumption of wild game contributing 500 μg/meal. Lastly, we found a strong association between BLLs and mercury exposure.

**Interpretation:**

Consumption of wild game hunted with lead ammunition may pose a common source of lead exposure in the Amazon. Communities that rely on wild game and wild fish may face a dual burden of exposure to lead and mercury, respectively.

## Introduction

Lead exposure is classified among the top ten chemicals of major global health concern due to its widespread presence in the environment and its cumulative and often irreversible health impacts on multiple organ systems. The use of lead in industrial products such as gasoline, paint, and piping have been largely phased out globally^1,2^; however, sources remain from environmental legacy pollution,^3^ industrial (e.g., oil extraction^4^) and behavioural (e.g., hunting with lead bullets^5^) factors that contribute to elevated lead exposures. Our study evaluated risk factors for lead exposure in Madre de Dios, Peru where oil exploitation, leaded paint and gasoline are unlikely confounders, which may allow us to better evaluate the relationship between lead exposure from consuming wild game.

In remote regions of the Amazon, there is increasing evidence of lead exposure; however, its principal source is still unknown. The lack of exposure to leaded paint^6^ and lead pipes, as well as relatively limited gasoline use, have led epidemiological studies to focus primarily on produced waters from oil extraction and oil spills. However, Anticona et al. studied blood lead levels (BLL) in communities impacted by oil spills in the Peruvian Amazon and found no difference in BLL between impacted and non-impacted communities.^4,7^ Although the oil industry is a source of environmental contamination,^8,9^ studies in the Amazon have not identified a significant relationship between oil extraction/spills with BLL.^4,7^

An isotopic analysis of hunted wild game in regions with and without oil extraction demonstrated that 86% and 57% of lead, respectively, could be traced to lead bullets.^10^ A bullet, upon impact, fragments into hundreds of pieces with 34% being under 0.01 g and are often microscopic, making it infeasible to remove all the lead from wild game.^11^ Hunting as a source of lead exposure has not been well studied in the Amazon, although lead exposure from hunting has been found in the Northern Hemisphere.^12,13^ Hunting with lead ammunition is prevalent throughout the Amazon, potentially making it a common, yet understudied, source of lead exposure.

Many indigenous and rural communities rely on wild game as a food source. In the 1960s, a study by Pierret and Marc of 430 households in the Northern Peruvian Amazon found 83% of them ate wild game at least once a week with an estimated 52 g of wild game consumed daily/capita.^14^ Recent studies have estimated an average of 63 kg/capita/year of wild game are consumed in rural and indigenous communities across the Amazon, equating to 1.3 million tons consumed annually.^15^ In 2012, Anticona et al. found 76.9% of indigenous study participants in Northern Peru ate wild game at least once per week.^16^ Although wild game consumption is highest in rural regions,^17^ consumption in urban communities continues throughout South America.^15,18^

**Figure 1.**
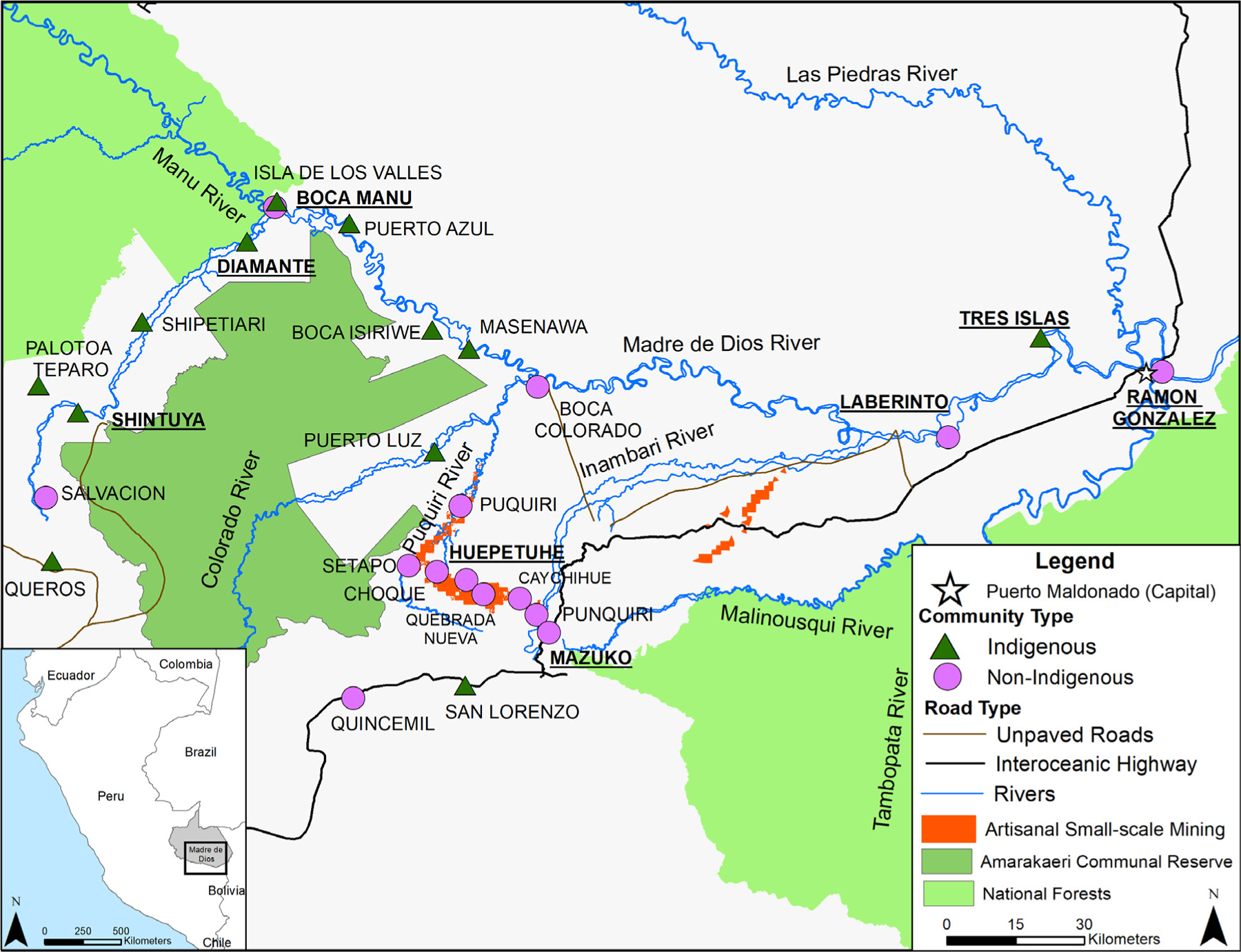
Map of study sites in Madre de Dios, Peru with indigenous and non-indigenous communities portrayed as green triangles and purple circles, respectively. Mining regions are depicted as orange, while national forests, including the Amarakaeri Communal, are green. The interoceanic highway is shown in black, while dirt roads that may become impassable during parts of the year are brown. Bold and underlined community labels are communities visited in 2018 and are part of the data subset.

The goal of this study is to evaluate risk factors of lead exposure in the Southern Peruvian Amazon region of Madre de Dios where there is currently no gas/oil exploitation. Previous studies in the region have shown elevated mercury exposure from fish consumption^19,20^ but no studies have focused on lead exposure. Indigenous communities predominantly live along the Madre de Dios River with limited or no road access (Figure 1). While several studies in the Amazon mention the potential for lead exposure from hunting,^16,21^ this is one of the few to assesses wild game consumption as a potential dietary risk factor for lead exposure.

## Methods

### Data collection

Data were collected in Madre de Dios, Peru in 2016 and in 2018 as part of the Amarakaeri Communal Reserve (ACR) and the Aetiology of Anaemia and Trace Metals (EATM) studies, respectively (Figure 1). Both studies were approved by the Institutional Review Board of La Universidad Peruana de Cayetano Heredia (IRB: 00001014 and 102134, respectively). The ACR study was designed to evaluate population level health factors such as anaemia^22^ and trace metal exposures and is described in Weinhouse et al. and Weinhouse et al. Briefly, indigenous, and non-indigenous communities surrounding the Amarakaeri Reserve were selected to measure the impact of gas exploration and gold mining. In communities of over 75 households, households with a woman between the ages of 15−49 years old (women of child-bearing age - WCBA) were randomly selected, while in smaller communities, all households with a WCBA were invited to participate. Roughly 10% of the study population were households without a WCBA. Blood data analysis from Weinhouse et al. focused on the collection of mercury with no lead data reported. The blood analysis was re-performed on all indigenous blood samples and a random selection of non-indigenous blood samples. A total of 295 blood samples were re-run in the Heileen Hsu-Kim laboratory at Duke University with a final of 245 individuals with complete data.

The EATM study communities were selected based on community type (upriver from gold mining, gold mining communities and urban communities), previous participation in Duke University studies, and logistical feasibility. Households were selected based on the anaemia status of children under 12 and their mothers, with families invited to enrol if either the child or mother had anaemia. Children and mothers were screened for anaemia at health posts, schools, and community centres by measuring haemoglobin using a HEMOCUE 201+, with written parental consent obtained prior to testing.^23^ Haemoglobin thresholds to classify anaemia status were dependent on age and sex as set by the World Health Organization.^24^ Enrolled families were administered a similar survey to the ACR study, but with additional information on food portion sizes and food sources. Household drinking water samples were also collected to measure lead concentrations (please refer to Supplementary information file for methods and results from water samples).

Data collection in both studies included anthropometrics (height, weight, body mass index [BMI]), household surveys, haemoglobin measurements taken with a HEMOCUE 201+, and a hair sample for total hair mercury analysis, a biomarker representing methylmercury exposure from fish consumption.^25^ The ACR study collected whole blood samples from adults (≥18 years of age), whereas the EATM study collected whole blood samples from all family members, including children, to be processed for trace metals. Survey administration, sample collection and sample processing were the same across studies.

### Laboratory methods

#### Blood:

Sampling methods for the ACR study are explained in detail in Weinhouse et al. ^20^ and were also followed in the EATM study. Briefly, whole blood samples were collected in Trace Metal free EDTA BD Vacutainer tubes and frozen on dry ice in the field to be analysed for mercury and lead. Blood samples were transported to Duke University on dry ice and stored at −80 °C until being processed in Dr Heileen Hsu-Kim’s laboratory at Duke University (Durham, North Carolina, USA).

To quantify lead and mercury levels, blood samples were thawed overnight at 4 °C and digested at ambient pressure on a hot block (Environmental Express, Charleston, South Carolina) at 65 °C. The digestions used ultra-trace clean digestion tubes (Environmental Express) and consisted of heating 0.5 ml of blood with 1 ml of 70% HNO_3_ (Plasma Pure Plus, SCP Science) and 0.05 ml of 30% HCl (Plasma Pure Plus, SCP Science) for two hours. The samples were cooled, 1 ml of 30% hydrogen peroxide (Plasma Pure Plus, SCP Science) was added to the mixture, and samples were heated again for 1 hour. After cooling, 10 μl of a 4 mg/L gold+2% HCl solution was spiked into the digestate to aid in mercury stability.

Each batch of 25 blood digestions included three blank samples, a NIST whole blood standard reference material (SRM, either 955c level 4 or 955d level 2), an aqueous standard (High Purity Standards, certified reference material-trace metals in drinking water Mix A (CRM-TMDW-A) + Spex Certiprep Hg), and an IAEA dry blood sample (IAEA-A-13). Two samples in each batch were analysed in triplicate. The relative standard deviation (%RSD) for triplicate digestions (*n* = 32) were 8.8% for Hg (range 0.8−32%) and 11.1% for Pb (range 0.4−36%) (See Supplemental Table 1). All samples with complete survey data were included in the analysis. Removing the 4 samples with triplicate Pb RSDs above 15% showed no difference in the analysis (Supplemental Table 2). We therefore include all of the data and report them for transparency purposes.

The blood digestates were diluted (10-fold) into an acid matrix (2% (v/v) HNO_3_ and 0.5% HCl (v/v)) containing 20 μg/L Au. Internal standards (^193^Ir and ^209^Bi) were also spiked into the matrix to correct for instrumental drift or matrix effects. The analyses were performed by Inductively Coupled Plasma-Mass Spectrometry (ICP-MS: Agilent 7900) in helium mode to reduce any potential for polyatomic interferences. The ICP-MS was tuned to reduce oxide interferences to less than 2%. The instrument was calibrated with purchased aqueous standards for Hg (Brooks Rand) and Pb (Spex Certiprep mix 2A), and the standard curves were verified with a secondary standard (High Purity Standards (CRM-TMDW-A) + Spex Certiprep (Hg)). Calibration verification checks were performed every 20 samples during a batch run. Mercury recoveries in were 95% for SRM 955c level 4 (*n* = 5), 83% for SRM 955d level 2 *n* = 9, and 99.6% for the aqueous Hg spike (*n* = 16). There are no reported values for mercury with IAEA-A-13. Lead recoveries in were 105% for SRM 955c level 4 (*n* = 5), 101% for SRM 955d level 2 (*n* = 9), 107% for the aqueous Pb spike (*n* = 16), and 80.4% for IAEA-A-13 (*n* = 16). Sample measurements were accepted if the corresponding NIST SRM 955c were 75−125% of the certified value for Hg and 85–115% of the certified value for Pb. The individual batch results for each NIST SRM 955c digestion are shown in Supplemental Table 3. The blanks for Hg were an average of 0.06 ug/L and for Pb were 0.1 ug/L.

#### Hair:

Hair samples were attached to self-adhesive notepaper, stored individually in paper envelopes, transported to Duke University, and stored at ambient temperature. The processing of hair samples is explained in detail in Weinhouse et al.^22^ Briefly, hair segments from the 2 cm closest to the root were analysed for total Hg content by direct combustion gold amalgamation atomic absorption spectrometry (Milestone Direct Mercury Analyzer 80), reflecting the last two months of exposure.^25^ The instrument was calibrated with aqueous Hg^2+^ acidified with 1M nitric acid, and calibration was verified by the analysis of a hair certified reference material (DB001, European Reference Materials) once per 10 hair samples in the analysis batch. Sample measurements were accepted if the corresponding reference material measurements were within 10% of the certified mean value. The lower limit of quantification was 1 ng total mercury (approximately 0.05 *μ*g/g in hair).

### Statistical analysis

Data from the ACR (*n* = 245) were pooled with participants from the EATM study (*n* = 62) to increase the sample size of non-native participants and incorporate a broader age range (Supplemental Table 4). Since anaemia may increase lead absorption, we used linear regression models to evaluate whether haemoglobin levels were associated with increased BLL (Supplemental Tables 5 and 6, *n* = 249, due to missing data in the pooled dataset). A sub-analysis was also conducted with only EATM data (*n* = 123) to evaluate risk factors that were not included in ACR study (increasing sample size), consisting of the risk factor of interest while adjusting for sex (Supplemental Table 7). *T*-tests and Fisher’s tests were used to identify potential risk factors of lead exposure and assess for differences between indigenous/non-indigenous participants and between the two studies. Pearson correlations were used to test correlations of mercury concentrations in hair and blood and blood lead levels in the pooled dataset. Linear mixed effect models with community as a random effect were used to evaluate risk factors for lead exposure. Blood lead levels were analysed as continuous and as categorical (high/low), with the United States’ Centre for Disease Control and Prevention (CDC) threshold of 5 μg/dL being used to differentiate between low vs. high exposures.^26^ The CDC has since lowered the blood lead level threshold to 3.5 μg/dL. Lead exposure was also assessed using ACR data to ensure similar results with and without pooling study data (results not shown). Risk factors were first tested individually, adjusting for sex; significant variables were subsequently tested together. Models were tested for heteroskedasticity and multicollinearity. Interactions between final risk factors and study were also evaluated to ensure differences in study design did not have a significant interaction on identified risk factors. Models were compared using the Bayesian Information Criterion (BIC) with the lowest BIC representing the best fit model. We use a classification and regression tree (CART) model, that uses recursive modelling techniques and residual sum of squares in a regression model to identify important variables and cut-off points to determine the ranking of risk factors to identify individuals with higher BLLs. In doing so, we create a classification tree of risk factors (indigenous status, age, sex; and consumption of yuca, fish, and wild game) and BLLs.

Survey data included participant and household information: demographics; smoking status (Yes/No); water source (Treated: municipal water/Untreated: river water, rainwater, well water); cooking fuel (Low emissions: natural gas, electric/High emission: wood, coal); and food frequency consumption of wild game, beef, chicken, and fish (Rarely/Monthly/Weekly). Wild game and fish were also evaluated binomially (Never or Weekly/Monthly and Weekly or non-Weekly), respectively. Age was categorized as adult/child (≥18 years old) and by quartiles for analysis. A variable for study (ACR/EATM) was also evaluated to test whether study design was a significant factor in the analysis. Community variables such as presence of food market (Yes/No), and paved access to the Interoceanic Highway (Yes/No) were also evaluated. Reference variables for all risk factors was either null, never, or low for ease of model interpretation.

The All-Ages Lead Model (AALM), Version 2.0, created by the United States Environmental Protection Agency (EPA), was used to determine whether periodic consumption of small bullet fragments (<0.01 g) in wild game could yield measured blood lead levels.^27^ The AALM is a pharmacokinetic model that simulates life-time lead exposure and predicts lead levels in multiple human tissues based on assumptions of age, sex, and exposure to environmental sources of lead. We apply this model by assuming wild game consumption is the predominate environmental lead source. We used default model settings and limit lead exposure to food using the background exposure at age three and pulse exposures to represent wild game consumption at age 10. Frequency of wild game consumption was estimated to be every 9 and 14 days for weekly and monthly consumption, respectively. Blood lead levels of individuals who never ate wild game were used to identify a background lead exposure. An estimated lead dose from wild game was then identified based on mean blood levels and frequency of wild game consumption with dose being held constant. A steady consumption of wild game, as stated in participants’ survey response, was assumed, and no other predominant sources of lead exposure were considered. We compared the AALM results to our pooled data by using nonparametric splines of blood lead concentrations by age, with 10 degrees of freedom, for each frequency of wild game consumption. We then used Pearson correlations to compare the AALM and spline models. Due to the potential of lead exposure from the use of lead fishing weights and yuca consumption (also known as manioc or cassava), we compared blood lead levels between fishermen and consumers of wild game (monthly or weekly) and evaluated yuca consumption in the EATM dataset (Supplemental Table 7, *n* = 123). In the same dataset, we evaluated blood lead and the amount of yuca, and wild game consumed. Blood lead, blood mercury and total hair mercury values were log10 transformed to normalize the data. All analyses were done in RStudio Version 1.2.5033.

### Role of funding source

Funding sources were not involved in study design, data collection, data analysis, writing or the submission process.

## Results

The final pooled data set included 307 participants with a mean age of 32 years, 65% female, and 54.6% native (Table 1). Between the two studies, the ACR study had older participants (35.2 years old vs. 22.6 years old, *p* < 0.0001); a larger proportion of men (0.37 vs. 0.23, *p* = 0.02) and indigenous participants (0.52 vs. 0.19, *p* < 0.0001); and participants with higher BLLs (3.8 μg/dL vs 2.1 μg/dL, *p* < 0.0001, Supplemental Table 4). Participants in the EATM study had significantly lower haemoglobin levels (12.2 vs 13.6, *p* < 0.0001, *n* = 249) and a higher prevalence of anaemia (0.5 vs. 0.0, *p* < 0.0001, *n* = 249, Supplemental Table 5). Haemoglobin was not associated with increased blood lead levels (Supplemental Table 6).

### EATM data subset

In the EATM sub-analysis (*n* = 123), larger portions of wild game were associated with higher BLLs. Individuals who ate one portion/serving had a 1.32 μg/dL (95% CI: 0.99−1.78) increase in BLLs, while those who ate two portions per serving had an increase of 1.66 μg/dL (95% CI: 1.10−2.57), compared to those who did not eat wild game (Table 2). No association was found between BLL and yuca consumption. All household water samples were below EPA’s guideline of 15 μg/L (Supplemental Figure 1).^26^

### Pooled data set

Risk factors were significantly different for individuals living in indigenous and non-indigenous communities. Indigenous communities were more likely to rely on untreated water and high emission fuels, compared to non-indigenous households. Non-indigenous individuals were predominantly male and younger than indigenous individuals. Overall, 18% of participants smoked, with smoking more prevalent among indigenous (Table 1). Protein sources varied significantly between indigenous and non-indigenous participants. Chicken was consumed most frequently. Beef was more frequently consumed by non-indigenous participants, and wild game by indigenous participants. 63% of non-indigenous participants never ate wild game, whereas 27% of indigenous participants ate it weekly and 66% monthly (Table 1).

Indigenous participants had significantly higher BLLs with an average of 5.7 μg/dL compared to 1.6 μg/dL (*p* < 0.0001, Table 3). Mean blood mercury and total hair mercury were nearly twice as high in indigenous compared to non-indigenous participants (Table 3). When blood lead and mercury exposure were categorized, 46.8% of indigenous participants had high lead (≥ 5.0 μg/dL) and mercury (≥ 5.8 μg/L) levels. Only 8.6% of indigenous participants had low blood lead and low mercury levels, compared to 51.8% of non-native participants (Table 1). Significant Pearson’s correlations of 0.37 (*p* < 0.0001) and 0.38 (*p* < 0.0001) were found between BLL and mercury levels in blood and hair, respectively.

In random effect models adjusting for sex, higher BLLs were associated with indigenous status, higher frequency of wild game consumption, male sex, blood mercury, smoking and lower beef consumption (Tables 4 and 5). Community as a random effect was significant, accounting for 12.5% of the variance at the community level. Being indigenous and male was associated with a 2.52 μg/dL (95% CI: 1.95−3.24) and 1.38 μg/dL (95% CI: 1.20−1.58) increase in BLLs, respectively (Table 4). Eating wild game monthly or weekly were both significantly associated with BLL, with weekly consumption having a larger effect on BLL (monthly: 1.41 μg/dL, 95% CI: 1.19−1.65, weekly: 1.70 μg/dL, 95% CI: 1.33−2.16). Modifying wild game consumption to weekly/monthly vs. never, improved model fit, resulting in wild game consumption being associated with a 1.42 μg/dL (95% CI: 1.21−1.68) increase in BLLs (Table 4). Similar results were found when analysing only ACR data (Supplemental Table 8). Smoking was associated with higher BLLs, while beef consumption, access to markets and the highway were associated with lower BLLs but reduced model fit. Age, BMI, water source, cooking fuel, haemoglobin, and study were not associated with lead exposure.

**Figure 2.**
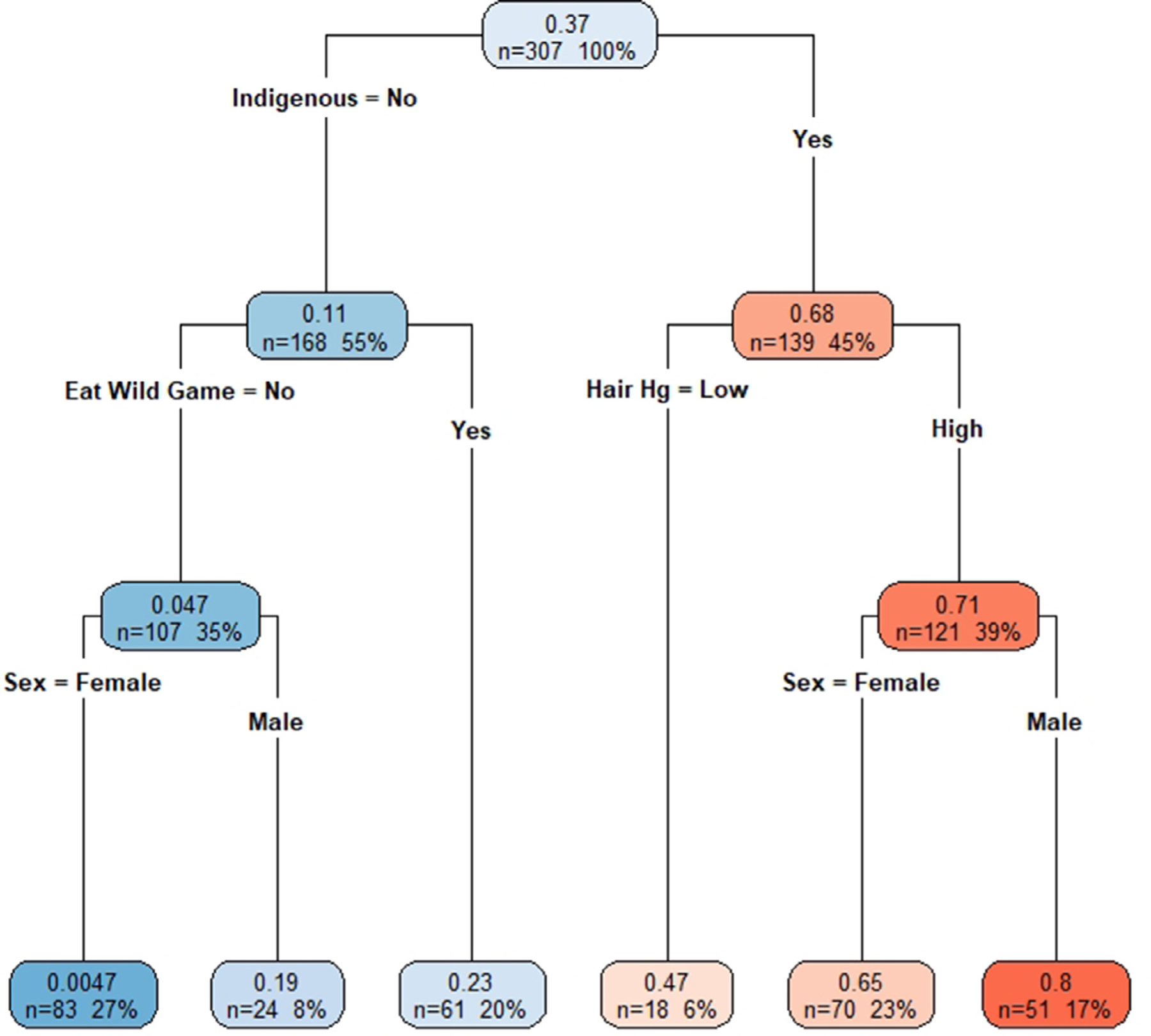

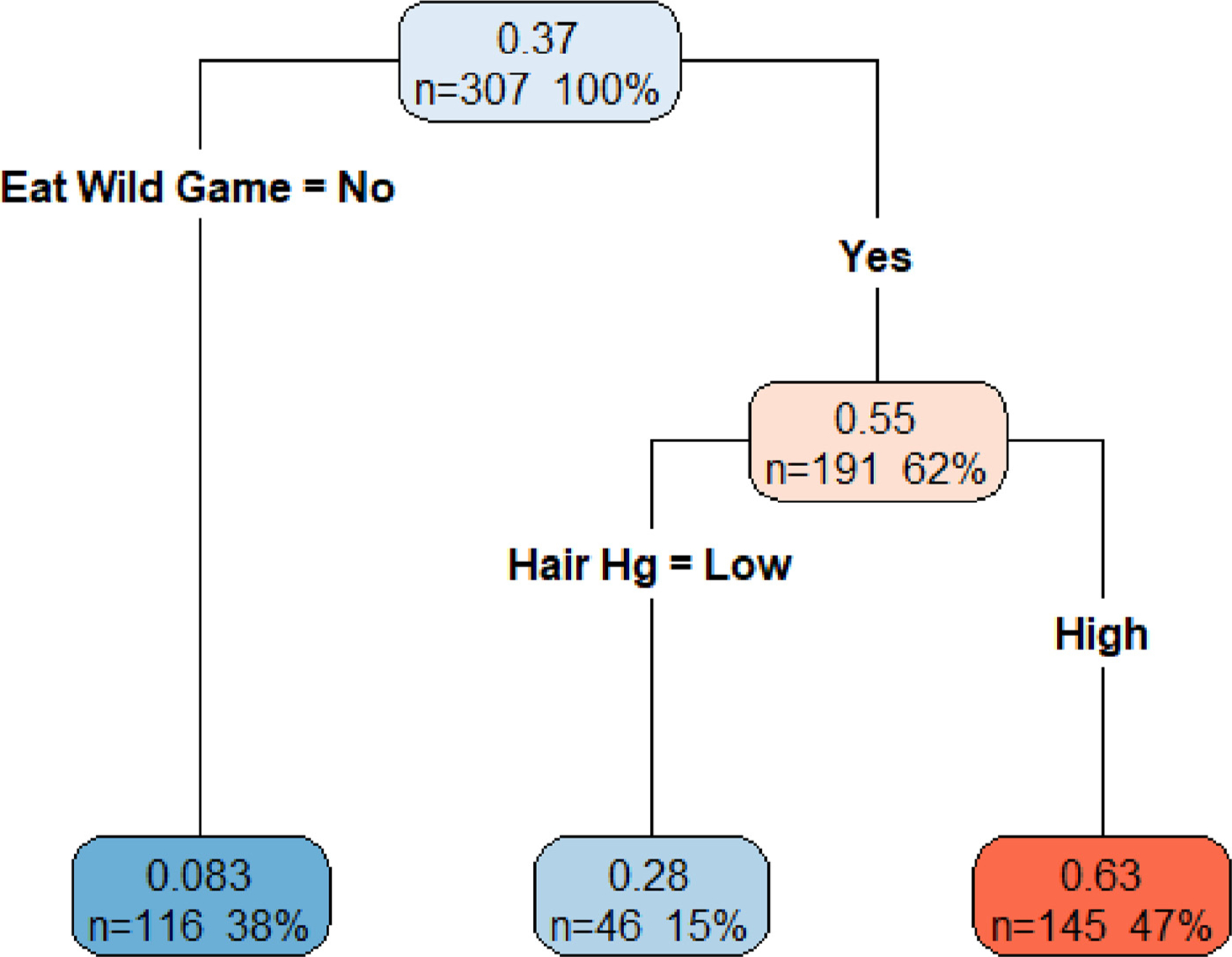
a (top figure). Colour coded classification tree, using the pooled data set, of log10 (BLL) with higher BLL represented with darker shades of red and lower BLL shown as darker shades of blue. In each box, top number is the predicted log10 (BLL) with bottom two numbers representing the number of participants found within the category and its proportion of the total study population, respectively. b (bottom figure). Colour coded classification tree, using the pooled data set, of log10 (BLL), with indigenous status excluded, with higher BLL represented with darker shades of red and lower BLL shown as darker shades of blue. In each box, top number is the predicted log10 (BLL) with bottom two numbers representing the number of participants found within the category and its proportion of the total study population, respectively.

With the CART model, we found indigenous status to be the greatest risk factor, followed by wild game consumption, fish consumption and sex (Figure 2a). Within indigenous communities, participants with high total hair mercury levels (>2 μg/g) had higher BLL. In non-indigenous communities, participants who ate wild game had higher BLLs, compared to those who did not (Figure 2a). Men had higher BLLs than women, regardless of community type. When excluding indigenous status, sex was no longer significant and wild game consumption was the main indicator of BLLs (Figure 2b).

**Figure 3.**
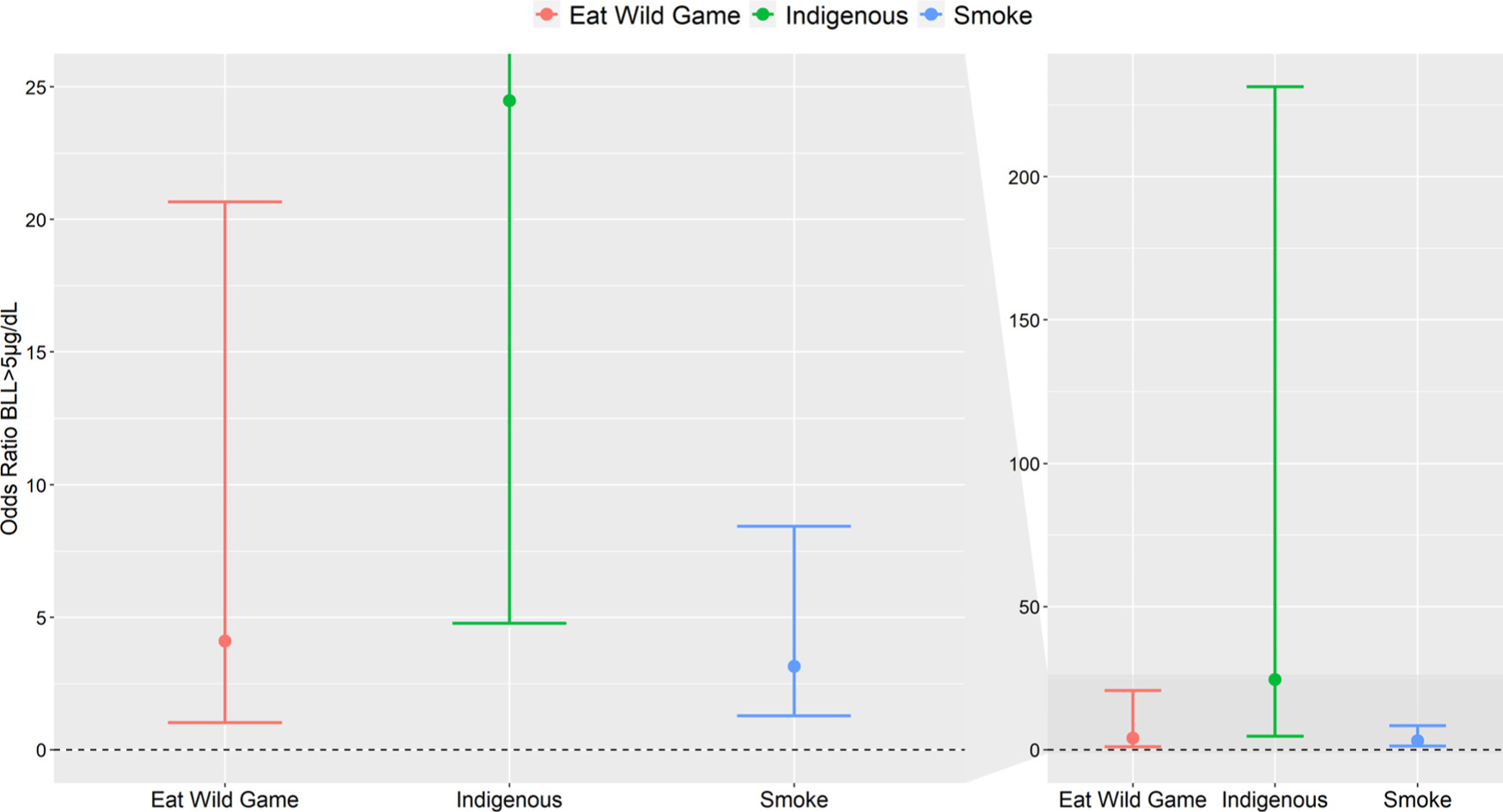
Odds ratio of best fit logit random effects model, using the pooled data set, for high blood lead levels (>5 μg/dl) that adjusts for wild game consumption, indigenous status, and smoking with community as a random effect. The split panel on the left is a zoomed in version of the panel on the right.

**Figure 4.**
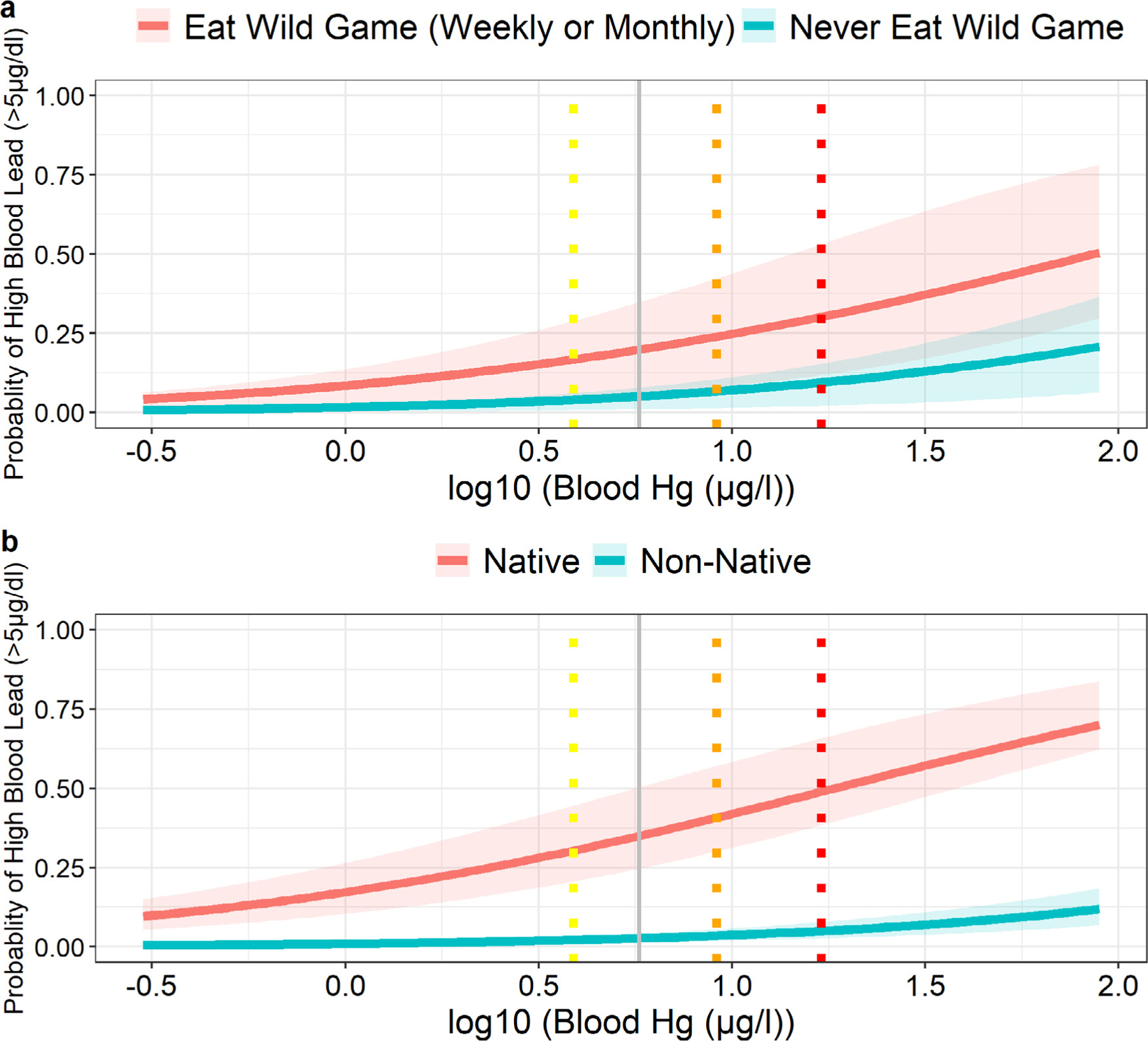
Probability of a blood lead level above the recommended guidelines of 5 μg/dl adjusting for log10 blood mercury and community as a random effect, using the pooled data set. **Panel A** also adjusts for wild game consumption, while **panel B** adjusts for native status. Dotted vertical lines represent the 25th, 50th and 75th percentiles of log10 blood mercury levels, shown as yellow, orange, and red, respectively. The solid grey line is the recommended limit of mercury in blood, 5.8 μg/l.

We also evaluated risk factors associated with BLLs above 5 μg/dL. Individually, high BLLs were associated with blood mercury, total hair mercury, smoking, sex, wild game consumption and indigenous status (Table 5). Indigenous participants had 24 times higher odds of having high BLLs than non-native participants, while those who ate wild game had four times higher odds than non-wild game consumers (Table 5, Figure 3). A strong association between BLLs and blood mercury levels was found, with a predicted 50% and 32% of indigenous participants and those who ate wild game (weekly or monthly), respectively, having high BLLs at the 75^th^ percentile of mercury exposure (Figure 4).

**Figure 5.**
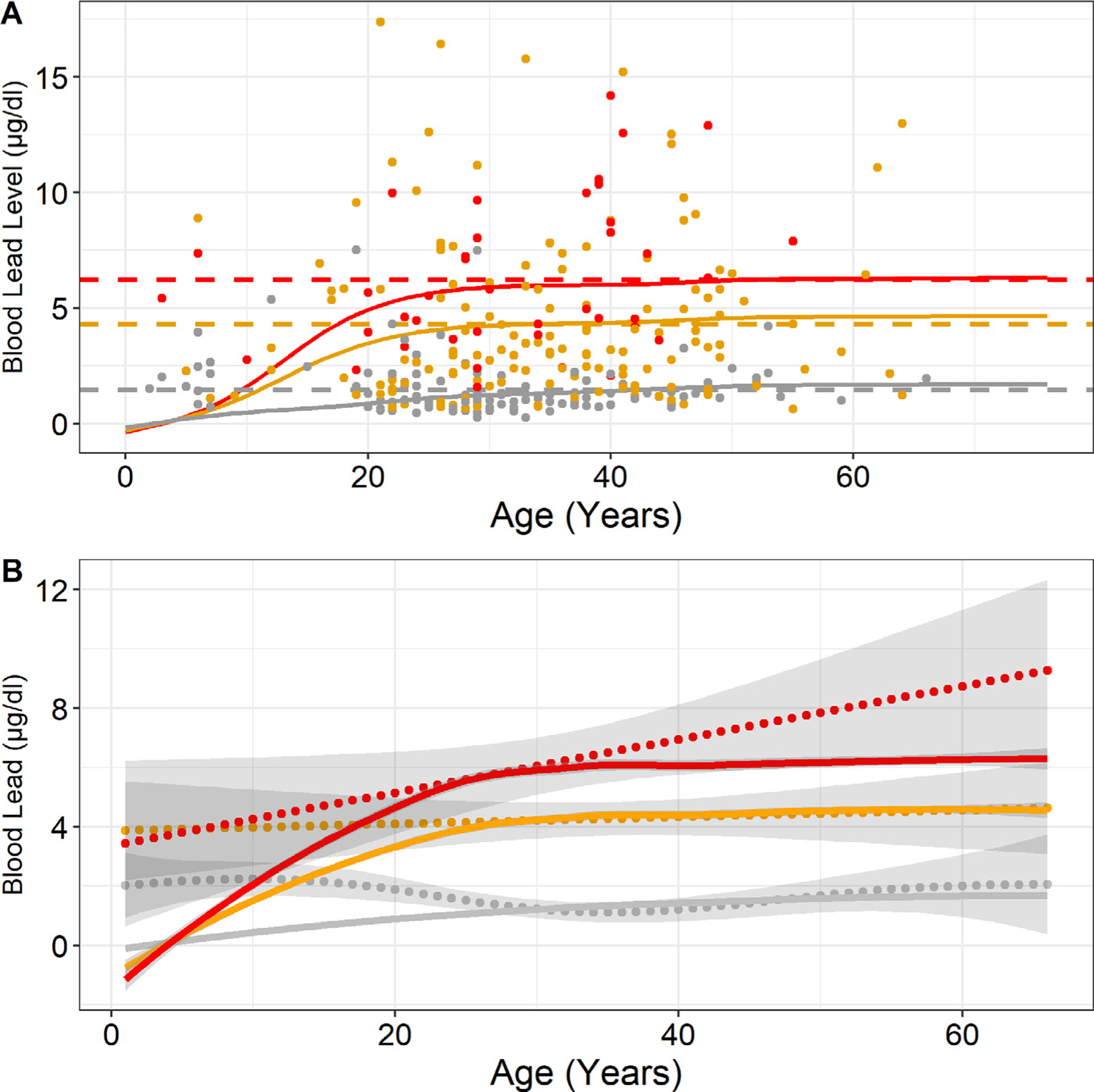
Modelled blood lead levels (solid lines) using All-Age Lead Model for baseline (no wild game consumption), monthly game consumption and weekly game consumption, shown in grey, yellow, and red, respectively (**Panel A**). Baseline model lead exposures consist of a 20 μg/day background exposure starting at age three. Lead exposure with consumption of wild game includes the 20 μg/day background and 500µg/day every 9 days or 14 days for weekly and monthly wild game consumption, respectively. Horizontal dotted lines demonstrate the mean blood lead levels in the study population by wild game consumption (Grey-Never (mean: 1.50 μg/dl, 95% CI: 0.48−4.43), Yellow-Monthly (mean: 4.27 μg/dl, 95% CI: 0.74−13.53), Red-Weekly (mean: 6.21 μg/dl, 95% CI: 2.06−12.91)). Points represent pooled study data (*n* = 307) shown by frequency of wild game consumption. **Panel B** compares the EPA AAL model (solid lines) shown in panel A with nonparametric splines (dotted lines with 95% confidence intervals) of measured blood lead levels by frequency of wild game consumption. Pearson correlations between spline models and EPA AAL models were −0.46 (*p* = 0.001), 0.83 (*p* < 0.001), and 0.82 (*p* < 0.001) for never, monthly, and weekly consuming wild game.

Blood lead exposure modelling using the AALM demonstrated that periodic lead exposures of 500 μg/meal of wild game and a background lead exposure of 20 μg/day replicated mean BLLs measured in the study (Figure 5, Panel A). The model assumes an estimated 500 μg of lead are ingested at each meal where wild game is consumed, which correlates with the fragmentation of bullets once they impact wild game. The AALM was significantly correlated with nonparametric splines of the pooled dataset with Pearson correlation values of 0.83 (*p* < 0.001), and 0.82 (*p* < 0.001) for monthly, and weekly wild game consumption, respectively (Figure 5, Panel B). The AALM and spline model for never have consumed wild game were inversely correlated when evaluating ages 0−65 (−0.46, *p* = 0.001); however, they were positively correlated above the 10th percentile or age 17 (0.28, *p* = 0.05) (Figure 5, Panel B).

## Discussion

This study found a strong indication, in a region without confounding lead sources of oil extraction, leaded paint, and leaded gasoline, that hunting is the predominant source of lead exposure, resulting in a 1.41 ug/dL higher BLL compared to individuals who do not consume wild game. This study also associates both increased frequency and larger portion sizes of wild game to higher BLLs, which has not yet been demonstrated in Madre de Dios or in the Peruvian Amazon overall. Using CART models, we identify indigenous communities as most at risk with male indigenous having the highest BLL of any subpopulation. Furthermore, the AALM model demonstrates that measured BLLs and frequency of wild game consumption (monthly and weekly) match, with an estimated dose of 500 μg Pb/meal and closely correlate with nonparametric splines of the pooled dataset (*n* = 307). For children under the age of 17, the AALM tends to underestimate BLL, likely due to our assumption in that there are no lead exposures prior to age 3. The estimated 500 μg/meal is reasonable with bullet fragments known to be microscopic and 34% less than 0.01 g.^11^ The evidence found in this study and the literature provides ample support that wild game consumption is an important route of lead exposure in hunting communities.

Results from the CART and logit models demonstrate that mercury and lead exposures are correlated, which may be due the use of lead fishing weights or the dependence of indigenous communities on wild fish and wild game as protein sources. Lead exposure is predominantly via ingestion and inhalation as lead absorption from handling is low. The percent transfer from hand to mouth in adults is ~24% with an estimated average lead exposure of 15.5 μg after handling lead sinkers for 15 s.^28^ In our study, we find no association with age or age class and BLLs, which would be expected if lead exposure were from handling fishing weights as children have more frequent hand to mouth contact.^29^

Indigenous and rural communities that rely on natural resources to meet nutritional needs face a dual exposure burden of lead and mercury.^30^ Communities that depend on wild game or wild fish to meet nutritional needs have been found to alternate between fish or wild game, depending on which is easier to obtain. Thus, as one resource becomes scarcer, dependence shifts to that which is more prevalent, depending on cultural, and personal preferences.^31^ This is especially important as fishing and hunting have traditionally been and continue to be the two main sources of protein in the Amazon. It may also help explain the significant correlation between lead and mercury exposure. Future epidemiological studies in the region should consider evaluating both metals due to overlapping toxicological health effects.

It is important to note that this study has several limitations. The study is cross-sectional and cannot evaluate how lead exposures vary across time. Although we find a strong correlation between wild game consumption and BLLs, it is possible that other dietary exposures exist. Previous studies in Brazil have identified consumption of farofa, toasted yuca/manioc used as a topping, to be a potential source of lead exposure, finding elevated concentrations of lead in yuca.^21,32^ Yuca is a food staple, eaten boiled or fried, in Madre de Dios and may also be a source of lead exposure; however, lead levels in yuca are likely site specific depending on the local geology. For example, soils of clay licks have been found to have higher lead levels.^10^ Although we find no association between yuca consumption and BLLs in our sub-analysis, the sample size may be too small (*n* = 123). Fermented yuca is used to make masato, a traditional drink, which was not included in our survey, potentially limiting our ability to adequately determine yuca consumption. The EATM study design did not randomly select study participants; thus, these results may not be reflective of the overall population. The pooling of data can create biases in results; however, we found similar results when analysing the datasets separately.

Lead shot has been linked to the lead poisoning of wildlife; however, demand for lead-free ammunition is low even at comparable prices.^33^ Worldwide, hunting is a main source of lead in the environment, with an estimated 80% of lead in European soils being attributable to lead ammunition by 2030.^34,35^ Yet, very few studies on lead exposure have been conducted in the Amazon. Due to the ubiquitous use of lead ammunition, consuming wild game may be an underestimated source of lead exposure worldwide. The United Nations’ Convention of Migratory Species, of which Peru is a member, seeks to eliminate lead shot in hunting. Fulfillment of this objective will likely not only improve the health of wildlife, but also of the many indigenous and rural communities that rely on wild game to meet nutritional and cultural needs.

## Supplementary Material

1

2

3

4

## Figures and Tables

**Table 1:** Individual and household risk factors evaluated for lead exposure between non-native and native status of the pooled dataset.

	Non-Native (*n* = 168)	Native (*n* = 139)	*P*-value	Overall (*n* = 307)	
**Age (Years)**					
Mean (SD)	32.1 (12.1)	33.4 (12.3)		32.7 (12.2)	
Median [Min, Max]	31.5 [2.00, 66.0]	33.0 [3.00, 64.0]		32.0 [2.00, 66.0]	
Sex**			0.002		
Female	124 (73.8%)	78 (56.1%)		202 (65.8%)	
Male	44 (26.2%)	61 (43.9%)		105 (34.2%)	
BMI*			0.04		
Mean (SD)	27.8 (5.56)	26.7 (4.10)		27.3 (4.98)	
Median [Min, Max]	28.4 [13.7, 42.6]	26.6 [16.1, 35.8]		27.3 [13.7, 42.6]	
Smoke Status**			0.003		
No	148 (88.1%)	103 (74.1%)		251 (81.8%)	
Yes	20 (11.9%)	36 (25.9%)		56 (18.2%)	
Household Water Source***			<0.001		
Treated	141 (83.9%)	46 (33.1%)		187 (60.9%)	
Untreated	27 (16.1%)	93 (66.9%)		120 (39.1%)	
Cooking Fuel***			<0.001		
High Emissions	24 (14.3%)	74 (53.2%)		98 (31.9%)	
Low Emissions	144 (85.7%)	65 (46.8%)		209 (68.1%)	
**Education Levely†**					
Elementary	33 (19.6%)	25 (18.0%)	0.07	58 (18.9%)	
Middle School	31 (18.5%)	41 (29.5%)		72 (23.5%)	
High School	78 (46.4%)	61 (43.9%)		139 (45.3%)	
Advanced	26 (15.5%)	12 (8.6%)		38 (12.4%)	
Highway Access***			<0.001		
Highway	75 (44.6%)	0 (0%)		75 (24.4%)	
Non-Highway	93 (55.4%)	139 (100%)		232 (75.6%)	
Fish Consumption**			0.003		
Rarely	9 (5.4%)	24 (17.3%)		33 (10.7%)	
Monthly	86 (51.2%)	58 (41.7%)		144 (46.9%)	
Weekly	73 (43.5%)	57 (41.0%)		130 (42.3%)	
Beef Consumption***			<0.001		
Never	19 (11.3%)	24 (17.3%)		43 (14.0%)	
Monthly	64 (38.1%)	86 (61.9%)		150 (48.9%)	
Weekly	85 (50.6%)	29 (20.9%)		114 (37.1%)	
Daily	0 (0%)	0 (0%)		0 (0%)	
Chicken Consumption***			<0.001		
Never	0 (0%)	2 (1.4%)		2 (0.7%)	
Monthly	9 (5.4%)	35 (25.2%)		44 (14.3%)	
Weekly	140 (83.3%)	102 (73.4%)		242 (78.8%)	
Daily	19 (11.3%)	0 (0%)		19 (6.2%)	
Wild Game Consumption***			<0.001		
Never	107 (63.7%)	9 (6.5%)		116 (37.8%)	
Monthly	58 (34.5%)	93 (66.9%)		151 (49.2%)	
Weekly	3 (1.8%)	37 (26.6%)		40 (13.0%)	
Blood Lead (Pb) and Mercury (Hg) Exposure (Low/High) ***					
Low blood Pb and Hg	87 (51.8%)	12 (8.6%)			99 (32.2%)
High blood Hg (≥ 5.8 μg/L), low Pb	77 (45.8%)	60 (43.2%)			137 (44.6%)
High blood Pb (≥ 5.0 μg/dL), low Hg	1 (0.6%)	2 (1.4%)			3 (1.0%)
High blood Pb and Hg	3 (1.8%)	65 (46.8%)			68 (22.1%)

Fisher’s Exact Test for categorical and *T*-tests for continuous variables.

Significance:

*** <0.001

** 0.001

* 0.01

† 0.05.

All large markets are in non-native communities.

**Table 2:** Random mixed effect model results for EATM data subset of portions of wild game consumed/meal and log10 blood lead (μg/dL) levels with community as a random effect.

Risk Factor	Estimate	95% Confidence Interval
Sex (Ref: Female)	0.15**	0.06−0.25
Wild Game per Serving (Ref: None)		
Half a Portion	0.12	−0.08−0.33
1 Portion	0.11.	−0.02−0.24
>2 Portions	0.22*	0.05−0.41

Significance:

*** <0.001

** 0.001

* 0.01

† 0.05.

**Table 3:** Measured blood lead, blood mercury and total hair mercury by evaluated risk factors and overall of the pooled dataset.

		Blood Lead Level (μg/dL)		Blood Mercury Level (μg/L)				Total Hair Mercury (μg/g)			
	*n*	Mean (SD)	Median[Min, Max]	Mean (SD)	Median [Min, Max]			Mean (SD)		Median [Min, Max]	
**Overall**	307	3.47 (3.26)	2.23	12.5 (12.3)	9.40			3.34 (3.05)		2.61	
			[0.25, 17.4]		[0.300, 89.1]					[0.0026, 21.4]	
**Sex**											
Female	202	2.85 (2.76)	1.69	10.9 (10.0)	8.65			3.10 (2.84)		2.55	
			[0.25, 15.2]		[0.310, 89.1]					[0.0704, 21.4]	
Male	105	4.68*** (3.78)	3.28	15.8** (15.2)	11.7			3.79y (3.39)		2.91	
			[0.52, 17.4]		[0.300, 73.7]					[0.0026, 19.4]	
**Smoking Status**											
No	251	3.08 (2.94)	2.07	11.9 (11.9)	8.90			3.19 (3.03)		2.50	
			[0.25, 15.8]		[0.30, 89.1]					[0.0026, 21.4]	
Yes	56	5.22*** (3.99)	4.15	15.4y (13.5)	13.1			3.98† (3.10)		3.23	
			[0.81, 17.4]		[0.43, 68.4]					[0.09, 13.2]	
**Indigenous Status**											
Non-Native	168	1.60 (1.30)	1.21	9.23 (11.9)		5.10	2.34 (2.61)			1.43	
			[0.25, 8.89]			[0.300, 89.1]				[0.0026, 13.8]	
Native	139	5.73*** (3.48)	4.95	16.6***		14.1	4.54*** (3.11)			3.84	
			[1.02, 17.4]	(11.5)		[1.60, 73.7]				[0.433, 21.4]	
**Wild Game Consumption^1^**											
Never	116	1.50 (1.21)	1.11	8.73 (12.5)		4.95			2.11 (2.09)	1.33	
			[0.25, 7.51]			[0.600, 89.1]				[0.0026, 13.3]	
Monthly	151	4.27*** (3.50)	3.11	14.1*** (11.9)		11.4			3.62*** (2.75)	2.91	
			[0.62, 17.4]			[0.300, 73.7]				[0.0899, 13.8]	
Weekly	40	6.21*** (3.20)	5.48	17.7*** (9.76)		16.9			5.84*** (4.44)	4.78	
			[1.59, 14.2]			[2.50, 47.7]				[0.569, 21.4]	
**Fish Consumption^1^**											
Never	33	3.93 (2.57)	3.17	12.3 (12.3)		9.60			3.15 (2.55)		2.61
			[0.66, 12.1]			[0.74, 68.9]					[0.35, 12.6]
Monthly	144	3.46 (3.38)	2.15	12.8 (12.8)		8.42			3.52 (3.12)		2.79
			[0.52, 16.4]			[0.31, 73.7]					[0.0026, 19.4]
Weekly	130	3.37 (3.29)	2.14	12.4 (11.8)		10.3			3.18 (3.09)		2.32
			[0.25, 17.4]			[0.30, 89.1]					[0.17, 21.4]

Fisher’s Exact Test for categorical and T-tests for continuous variables

Significance:

*** <0.001

** 0.001

* 0.01

† 0.05.

1 ANOVA with consumption frequency of Weekly and Monthly compared to Never.

**Table 4:** Random mixed effect model results for individuals who eat wild game (weekly or monthly) and log10 blood lead levels (μg/dL) with community as a random effect, using the pooled dataset.

Risk Factor	Estimate	95% Confidence Interval
Sex (Ref: Female)	0.14***	0.08−0.20
Native (Ref: Non-indigenous)	0.40***	0.29−0.51
Eat Wild Game (Ref: Never)	0.15**	0.08−0.23

Significance:

*** <0.001

** 0.001

* 0.01

† 0.05.

**Table 5:** Odds ratios and 95% confidence intervals (CI) from random mixed effect logit models for high BLL levels (>5 μg/dL) with community as a random effect, using the pooled dataset.

	Univariate Models		Multivariate Model	
Risk Factor	Odds Ratio	95% CI	Odds Ratio	95% C.I.
				
Sex (Ref: Female)	3.28**	1.51−7.47		
Native (Ref: Non-indigenous)	42.02***	8.95−354.66	24.53***	4.77−231.34
Smoke (Ref: No)	4.02**	1.57−11.29	3.16*	1.28−8.44
Eat Wild Game (Ref: Never)	6.86**	1.78−33.55	4.10.	1.02−20.67
Wild Game Consumption (Ref: Never)				
Monthly	6.51*	1.67−32.08		
Weekly	8.44**	1.83−47.33		
Log10(Blood Hg)	17.14***	4.32−77.21		
Log10(Total Hair Hg)	8.08**	1.93−37.13		

Significance:

*** <0.001

** 0.001

* 0.01

† 0.05.

## Research in context

### Evidence before the study

In high-income countries, environmental lead exposure is often associated with soil contamination from the historical use of leaded gasoline and lead paint; however, risk factors of lead exposure in places like the Amazon are unclear. Potential lead sources include oil spills, lead fishing weights, yucca consumption, and hunting with lead ammunition. Studies with the First-Nation people of Ontario, Canada found consumption of hunted game to be a primary source of lead exposure due to the inadvertent consumption of microscopic lead fragments. Although few lead exposure assessments have been conducted in the Amazon, previous studies have found indigenous in the Amazon have the highest levels of mercury exposure due to higher fish consumption. We assess hunting as a source of lead exposure in the southeastern Peruvian Amazon region of Madre de Dios to determine if this could be also a contributing source of lead exposure.

### Added value of the study

We find increased consumption frequency and larger portion sizes of wild game to be associated with higher blood lead levels (BLL). Using the Environmental Protection Agency’s All Ages Lead Model, we estimated that a meal of wild game results in a lead exposure 25 times greater than background. We also identified indigenous participants to have the highest risk of lead exposure, likely due to higher frequency of wild game consumption. This finding shows that indigenous and rural communities in the region face a dual exposure burden to lead and mercury due to consumption of wild game and fish as primary protein sources.

### Implications of all the available evidence

Our study assesses multiple risk factors of lead exposure and provides further evidence of wild game consumption as a source of lead exposure. It also supports the long-held hypothesis that indigenous communities experience the gravest environmental injustices in the Amazon. High levels of both lead and mercury in these populations will result in significant neurocognitive impairments that jeopardize the sustainability of these communities. As wild game and wild fish consumption is prevalent in the Amazon, lead exposure will continue to impact these populations. Evidence-based interventions should prioritize the implementation of non-lead ammunition to reduce human lead exposure.
