# Supplementary material for: Risk of lead exposure from wild game consumption from cross-sectional studies in Madre de Dios, Peru
Source: Lancet Reg Health Am. 2022 May 8;12:100266. doi: 10.1016/j.lana.2022.100266 (PMC9555248; doi:10.1016/j.lana.2022.100266)
Supplement: Supplementary file 1 [file mmc1.docx]

**Supplemental Figures**

**Supplemental Figure 1.** Boxplots of lead levels in household water samples for each study community in the data subset. Blue circles represent lead levels of individual water samples. Solid black dots represent outliers. Indigenous communities are labelled with an asterisk. Samples were collected in pre-cleaned 125 mL HDPE bottles. Samples were transported on ice to the laboratory where they were acidified to 2% nitric acid and stored at 4°C prior to analysis. Lead concentration was determined using Inductively Coupled Plasma-Mass Spectrometry (ICP-MS: Agilent 7900) in helium mode to reduce any potential for polyatomic interferences. The lowest quantifiable limit for the analysis was 0.025 µg/L Pb.

**Supplemental Tables**

**Supplemental Table 1.** Whole blood mercury (Hg) and lead (Pb) concentrations for samples that were analysed in triplicate. Each digestion batch comprised 2 samples digested in triplicate. The data include the measured value for each replicate, their average, standard deviation (SD) and relative standard deviation (RSD) for each set of sample triplicates. Batch code is represented by capital letters.

**Supplemental Table 2**. Random mixed effect model results for individuals who eat wild game (weekly or monthly) and log10 blood lead levels (µg/dL) with community as a random effect, excluding triplicates with blood lead RSDs above 15% from the pooled dataset (n=303).

**Supplemental Table 3.** Analysis results for blood Standard Reference Material (SRM) purchased from the National Institute of Standards and Technology (NIST), Each digestion batch include one SRM that was processed in parallel with the study samples.

**Supplemental Table 4.** Descriptive statistics of the Amarakaeri Communal Reserve (ACR) and the Aetiology of Anaemia and Trace Metals (EATM) studies.

**Supplemental Table 5.** Descriptive statistics of ACR and EATM studies to evaluate blood lead levels and anaemia. Fisher’s Exact Test for categorical and T-tests for continuous variables were used to evaluate differences between the ACR and EATM study.

**Supplemental Table 6.** Random mixed effect model results to test whether haemoglobin is associated with increased blood lead in the pooled data set (n=249) with community as a random effect.

**Supplemental Table 7:** Descriptive statistics for the Aetiology of Anaemia and Trace Metals (EATM) study.

**Supplemental Table 8.** Random mixed effect model results for individuals who eat wild game (weekly or monthly) and log10 blood lead levels (µg/dL) with community as a random effect, using only the ACR dataset (n = 245).
