# Supplementary material for: Risk of lead exposure from wild game consumption from cross-sectional studies in Madre de Dios, Peru
Source: Lancet Reg Health Am. 2022 May 8;12:100266. doi: 10.1016/j.lana.2022.100266 (PMC9555248; doi:10.1016/j.lana.2022.100266)
Supplement: Supplementary file 2 [file mmc2.docx]

**Supplemental Information**

**Supplemental Methods**

*Drinking Water:*

For the 2018 study, a water sample was collected for each enrolled household by filling 125-mL pre-cleaned high density polyethylene bottles from the main faucet or water storage bucket of the household. Additional samples were collected from school tap water and community drinking water sources. Each bottle was labelled, stored in sealable plastic bags, held in cold storage with ice packs in the field, and frozen during international transit to and storage at Duke University. At Duke laboratories, water samples were thawed, acidified to a concentration of 2% v/v concentrated HNO_3_, held at 4°C, and analysed for Pb by ICP-MS (Agilent 7900). Analysis of Pb was carried out under a He atmosphere (collision cell) to reduce polyatomic ​interferences. All standards and samples were diluted into a 2% HNO_3_ (Fisher Scientific trace metal grade) matrix made with18.2 MΩ water prior to analysis. The signals for Pb isotopes ^206^Pb, ^207^Pb, and ^208^Pb were summed to obtain Pb concentrations. ^209^Bi was used as internal standards to correct for shifts in the analyte signal intensity during the analysis run. The calibration was verified using NIST traceable second source standard (CRM-TMDW-A). The lower limit of quantification was 0.03 µg/L.

**Supplemental Figures**

**
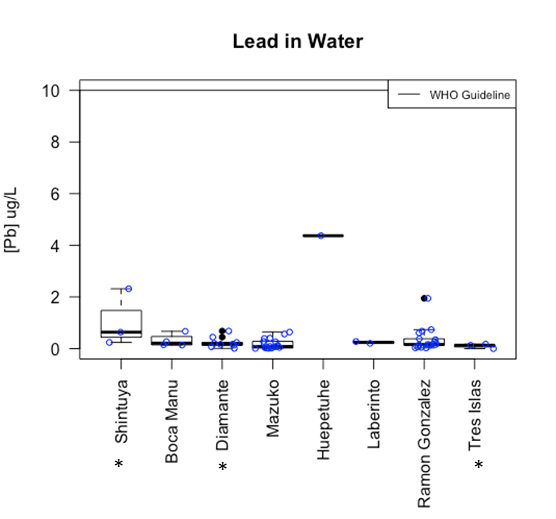
**

**Supplemental Figure 1:** Boxplots of lead levels in household water samples for each study community in the data subset. Blue circles represent lead levels of individual water samples. Solid black dots represent outliers. Indigenous communities are labelled with an asterisk. Samples were collected in pre-cleaned 125 mL HDPE bottles. Samples were transported on ice to the laboratory where they were acidified to 2% nitric acid and stored at 4°C prior to analysis. Lead concentration was determined using Inductively Coupled Plasma-Mass Spectrometry (ICP-MS: Agilent 7900) in helium mode to reduce any potential for polyatomic interferences. The lowest quantifiable limit for the analysis was 0.025 µg/L Pb.
