# Supplementary material for: Risk of lead exposure from wild game consumption from cross-sectional studies in Madre de Dios, Peru
Source: Lancet Reg Health Am. 2022 May 8;12:100266. doi: 10.1016/j.lana.2022.100266 (PMC9555248; doi:10.1016/j.lana.2022.100266)
Supplement: Supplementary file 3 [file mmc3.docx]

***Supplemental Tables***

**Supplemental Table 1:** Whole blood mercury (Hg) and lead (Pb) concentrations for samples that were analysed in triplicate. Each digestion batch comprised 2 samples digested in triplicate. The data include the measured value for each replicate, their average, standard deviation (SD) and relative standard deviation (RSD) for each set of sample triplicates. Batch code is represented by capital letters.

| Blood Digestion Batch | [Hg]_rep 1_ | [Hg]_rep 2_ | [Hg]_rep 3_ | [Hg]_average_ | [Hg]_SD_ | RSD | [Pb]_rep 1_ | [Pb]_rep 2_ | [Pb]_rep 3_ | [Pb]_average_ | [Pb]_SD_ | RSD |
| --- | --- | --- | --- | --- | --- | --- | --- | --- | --- | --- | --- | --- |
|  | µg/L | µg/L | µg/L | µg/L | µg/L | % | µg/L | µg/L | µg/L | µg/L | µg/L | % |
| CA triplicate 1 | 47.2 | 43.4 | 37.2 | 42.6 | 5.0 | 12 | 76.1 | 70.1 | 62.2 | 69.5 | 7.0 | 10.0 |
| CA triplicate 2 | 15.0 | 16.8 | 16.8 | 16.2 | 1.0 | 6.2 | 44.2 | 43.5 | 44.1 | 43.9 | 0.3 | 0.8 |
| AB triplicate 1 | 20.7 | 20.6 | 20.4 | 20.6 | 0.2 | 0.8 | 127.1 | 125.6 | 124.5 | 125.7 | 1.3 | 1.0 |
| AB triplicate 2 | 9.8 | 9.8 | 9.2 | 9.6 | 0.4 | 4.0 | 43.8 | 44.5 | 40.6 | 43.0 | 2.1 | 4.8 |
| AC triplicate 1 | 18.4 | 18.4 | 16.8 | 17.9 | 0.9 | 5.2 | 81.3 | 80.0 | 73.0 | 78.1 | 4.5 | 5.8 |
| AC triplicate 2 | 12.8 | 12.8 | 12.5 | 12.7 | 0.1 | 1.2 | 29.6 | 29.4 | 29.6 | 29.5 | 0.1 | 0.4 |
| AD triplicate 1 | 0.3 | 0.3 | 0.3 | 0.3 | 0.0 | 4.2 | 20.5 | 26.1 | 24.9 | 23.8 | 3.0 | 12.5 |
| AD triplicate 2 | 0.8 | 0.9 | 0.7 | 0.8 | 0.1 | 17 | 17.6 | 19.9 | 15.1 | 17.5 | 2.4 | 13.8 |
| AE triplicate 1 | 1.2 | 0.7 | 1.3 | 1.1 | 0.3 | 31 | 20.2 | 10.9 | 22.7 | 17.9 | 6.2 | 34.8 |
| AE triplicate 2 | 1.1 | 1.1 | 1.1 | 1.1 | 0.0 | 3.2 | 19.1 | 17.9 | 18.7 | 18.6 | 0.6 | 3.2 |
| AF triplicate 1 | 16.0 | 17.9 | 16.6 | 16.9 | 1.0 | 5.8 | 46.4 | 51.1 | 45.6 | 47.7 | 3.0 | 6.2 |
| AF triplicate 2 | 3.3 | 3.3 | 5.6 | 4.1 | 1.3 | 32 | 19.5 | 20.3 | 30.5 | 23.5 | 6.1 | 26.2 |
| AG triplicate 1 | 13.2 | 12.5 | 13.1 | 12.9 | 0.4 | 2.9 | 64.4 | 62.3 | 66.7 | 64.5 | 2.2 | 3.4 |
| AG triplicate 2 | 18.4 | 18.7 | 20.6 | 19.2 | 1.2 | 6.3 | 55.2 | 56.5 | 60.8 | 57.5 | 2.9 | 5.0 |
| AH triplicate 1 | 4.0 | 3.6 | 3.3 | 3.6 | 0.3 | 9.5 | 35.0 | 30.4 | 28.0 | 31.1 | 3.6 | 11.4 |
| AH triplicate 2 | 26.1 | 25.7 | 25.6 | 25.8 | 0.2 | 0.9 | 24.2 | 23.5 | 24.2 | 24.0 | 0.4 | 1.6 |
| AI triplicate 1 | 17.1 | 14.3 | 18.5 | 16.7 | 2.1 | 13 | 95.2 | 82.3 | 100.7 | 92.7 | 9.4 | 10.2 |
| AJ triplicate 1 | 14.7 | 17.4 | 15.0 | 15.7 | 1.5 | 9.5 | 14.0 | 15.9 | 14.1 | 14.7 | 1.0 | 7.1 |
| AK triplicate 1 | 69.6 | 64.8 | 70.9 | 68.4 | 3.2 | 4.7 | 4.2 | 2.8 | 3.3 | 3.4 | 0.7 | 21.1 |
| AK triplicate 2 | 21.5 | 19.1 | 26.2 | 22.3 | 3.6 | 16 | 6.6 | 3.2 | 4.7 | 4.8 | 1.7 | 35.7 |
| AL triplicate 1 | 7.5 | 6.6 | 6.5 | 6.9 | 0.6 | 8.3 | 5.7 | 5.2 | 4.9 | 5.3 | 0.4 | 8.2 |
| AL triplicate 2 | 2.0 | 2.7 | 2.8 | 2.5 | 0.4 | 17 | 6.7 | 8.6 | 8.4 | 7.9 | 1.0 | 13.0 |
| BA triplicate 1 | 6.9 | 7.0 | 7.1 | 7.0 | 0.1 | 1.5 | 60.1 | 48.8 | 53.5 | 54.1 | 5.7 | 10.5 |
| BA triplicate 2 | 13.9 | 14.8 | 14.7 | 14.5 | 0.5 | 3 | 72.0 | 76.8 | 76.5 | 75.1 | 2.7 | 3.6 |
| BB triplicate 1 | 5.8 | 5.9 | 5.9 | 5.9 | 0.1 | 1.5 | 9.0 | 9.7 | 10.0 | 9.6 | 0.5 | 5.4 |
| BB triplicate 2 | 1.8 | 1.7 | 1.8 | 1.8 | 0.0 | 2.5 | 32.0 | 33.2 | 33.1 | 32.8 | 0.6 | 2.0 |
| BC triplicate 1 | 4.6 | 4.5 | 4.7 | 4.6 | 0.1 | 2.1 | 57.8 | 57.2 | 63.1 | 59.4 | 3.3 | 5.5 |
| BC triplicate 2^*^ | 2.7 | 2.9 | 3.4 | 3.0 | 0.4 | 13.2 | 40.2 | 46.7 | 56.4 | 47.7 | 8.1 | 17.1 |
| BD triplicate 1^*^ | 13.0 | 15.2 | 20.2 | 16.2 | 3.7 | 23 | 17.5 | 22.4 | 34.1 | 24.7 | 8.6 | 34.6 |
| BD triplicate 2 | 4.2 | 4.2 | 4.3 | 4.2 | 0.1 | 2.2 | 14.8 | 15.2 | 15.5 | 15.1 | 0.3 | 2.2 |
| BF triplicate 1^*^ | 1.7 | 2.2 | 1.7 | 1.9 | 0.3 | 15.0 | 9.9 | 15.4 | 10.4 | 11.9 | 3.0 | 25.3 |
| BE triplicate 1 | 6.2 | 7.1 | 6.1 | 6.5 | 0.5 | 8.2 | 7.8 | 9.4 | 7.8 | 8.3 | 0.9 | 11.3 |
| Average |  |  |  |  |  | 8.9 |  |  |  |  |  | 11.1 |
| SD |  |  |  |  |  | 8.3 |  |  |  |  |  | 10.3 |
| Median |  |  |  |  |  | 6.0 |  |  |  |  |  | 7.6 |
| Minimum |  |  |  |  |  | 0.8 |  |  |  |  |  | 0.4 |
| Maximum |  |  |  |  |  | 32.3 |  |  |  |  |  | 35.7 |

^*^Not included in analysis due to missing survey data

**Supplemental Table 2:** Random mixed effect model results for individuals who eat wild game (weekly or monthly) and log10 blood lead levels (µg/dL) with community as a random effect, excluding triplicates with blood lead RSDs above 15% from the pooled dataset (n=303).

| Risk Factor | Estimate | 95% Confidence Interval |
| --- | --- | --- |
| Sex (Ref: Female) | 0.15*** | 0.09-0.21 |
| Native (Ref: Non-indigenous) | 0.41*** | 0.30-0.51 |
| Eat Wild Game (Ref: Never) | 0.15** | 0.08-0.22 |

Significance: <0.001 ‘ *** ’; 0.001 ‘ ** ’; 0.01 ‘ * ’; 0.05 ‘ † ’

**Supplemental Table 3**: Analysis results for blood Standard Reference Material (SRM) purchased from the National Institute of Standards and Technology (NIST), Each digestion batch include one SRM that was processed in parallel with the study samples.

| **SRM 955d^a^ Level 2** | | | **SRM 955c^b^ level 2** | | | **SRM 955c level 4** | | |  |
| --- | --- | --- | --- | --- | --- | --- | --- | --- | --- |
|  | Hg (µg/L) | Pb (µg/L) |  | Hg (µg/L) | Pb (µg/L) |  | Hg (µg/L) | Pb (µg/L) | |
| NIST Certified value | 6.83 | 49.47 | NIST Certified value | 4.95 | 139.5 | NIST Certified value | 33.9 | 455.3 | |
| Digestion batch code |  |  | Digestion batch code |  |  | Digestion batch code |  |  | |
| AC | 6.46 | 49.85 | BA | 4.80 | 142.4 | BA | 32.2 | 484.3 | |
| AD | 5.63 | 51.50 | CA | 4.64 | 158.2 | BB | 34.1 | 473.6 | |
| AE | 5.51 | 49.46 |  |  |  | BC | 30.5 | 455.1 | |
| AF | 5.63 | 49.94 |  |  |  | B_D/F* | 34.0 | 481.4 | |
| AG | 5.77 | 46.40 |  |  |  | BE | 29.9 | 492.8 | |
| AH | 5.98 | 53.21 |  |  |  |  |  |  | |
| AIAJ* | 5.68 | 51.24 |  |  |  |  |  |  | |
| AK | 5.69 | 46.92 |  |  |  |  |  |  | |
| AL | 5.53 | 50.21 |  |  |  |  |  |  | |
| Average | 5.8 | 49.9 | Average | 4.7 | 150.3 | Average | 32.2 | 477.4 | |
| Standard Deviation | 0.3 | 2.1 | Standard Deviation | 0.1 | 11.2 | Standard Deviation | 1.9 | 14.3 | |
| % RSD | 5.1 | 4.3 | % RSD | 2.4 | 7.4 | % RSD | 6.0 | 3.0 | |
| % Recovery | 84.4 | 100.8 | % Recovery | 95.4 | 107.7 | % Recovery | 94.9 | 104.9 | |
| a-NIST SRM is of whole human blood. b-NIST SRM of whole caprine blood *AI and AJ batches digested on same day. AJ batch was a partial batch so only one NIST standard digested for that day. BD and BF batches digested on same day. BF was a partial batch so only one NIST sample digested for that day. | | | | | | | | |  |
|  |  |  |  |  |  |  |  |  |  |

**Supplemental Table 4:** Descriptive statistics of the Amarakaeri Communal Reserve (ACR) and the Aetiology of Anaemia and Trace Metals (EATM) studies.

|  | EATM | ACR | P-value | Overall |
| --- | --- | --- | --- | --- |
|  | (N=62) | (N=245) |  | (N=307) |
| **Age (Years)** |  |  |  |  |
| Mean (SD) | 22.6 (12.4) | 35.2 (10.8) | <0.001 | 32.7 (12.2) |
| Median [Min, Max] | 25.0 [2.00, 49.0] | 35.0 [15.0, 66.0] |  | 32.0 [2.00, 66.0] |
| **Sex** |  |  | 0.02 |  |
| Female | 48 (77.4%) | 154 (62.9%) |  | 202 (65.8%) |
| Male | 14 (22.6%) | 91 (37.1%) |  | 105 (34.2%) |
| **Smoke Status** |  |  | 0.001 |  |
| No | 59 (95.2%) | 192 (78.4%) |  | 251 (81.8%) |
| Yes | 3 (4.8%) | 53 (21.6%) |  | 56 (18.2%) |
| **Indigenous Status** |  |  | <0.001 |  |
| Native | 12 (19.4%) | 127 (51.8%) |  | 139 (45.3%) |
| Non-Native | 50 (80.6%) | 118 (48.2%) |  | 168 (54.7%) |
| **Community Type** |  |  |  |  |
| Mining | 39 (62.9%) | 40 (16.3%) |  | 79 (25.7%) |
| Native | 12 (19.4%) | 127 (51.8%) |  | 139 (45.3%) |
| Urban | 11 (17.7%) | 78 (31.8%) |  | 89 (29.0%) |
| **Household Water Source** |  |  |  |  |
| Treated | 41 (66.1%) | 146 (59.6%) |  | 187 (60.9%) |
| Untreated | 21 (33.9%) | 99 (40.4%) |  | 120 (39.1%) |
| **Cooking Fuel** |  |  | 0.08 |  |
| High Emissions | 23 (37.1%) | 75 (30.6%) |  | 98 (31.9%) |
| Low Emissions | 39 (62.9%) | 170 (69.4%) |  | 209 (68.1%) |
| **Education level** |  |  | <0.001 |  |
| Advanced | 6 (9.7%) | 32 (13.1%) |  | 38 (12.4%) |
| Elementary | 24 (38.7%) | 34 (13.9%) |  | 58 (18.9%) |
| High school | 21 (33.9%) | 118 (48.2%) |  | 139 (45.3%) |
| Middle school | 11 (17.7%) | 61 (24.9%) |  | 72 (23.5%) |
| **Beef Consumption** |  |  | <0.001 |  |
| Never | 17 (27.4%) | 26 (10.6%) |  | 43 (14.0%) |
| Monthly | 11 (17.7%) | 139 (56.7%) |  | 150 (48.9%) |
| Weekly | 34 (54.8%) | 80 (32.7%) |  | 114 (37.1%) |
| **Chicken Consumption** |  |  | <0.001 |  |
| Never | 0 (0%) | 2 (0.8%) |  | 2 (0.7%) |
| Monthly | 3 (4.8%) | 41 (16.7%) |  | 44 (14.3%) |
| Weekly | 40 (64.5%) | 202 (82.4%) |  | 242 (78.8%) |
| Daily | 19 (30.6%) | 0 (0%) |  | 19 (6.2%) |
| **Wild Game Consumption** | |  | <0.001 |  |
| Never | 40 (64.5%) | 76 (31.0%) |  | 116 (37.8%) |
| Monthly | 16 (25.8%) | 135 (55.1%) |  | 151 (49.2%) |
| Weekly | 6 (9.7%) | 34 (13.9%) |  | 40 (13.0%) |
| **Blood Lead Level (µg/dL)** |  |  | <0.001 |  |
| Mean (SD) | 2.13 (2.14) | 3.81 (3.41) |  | 3.47 (3.26) |
| Median [Min, Max] | 1.22 [0.250, 9.97] | 2.45 [0.250, 17.4] |  | 2.23 [0.250, 17.4] |
| **Blood Mercury Level (µg/L)** | |  | <0.001 |  |
| Mean (SD) | 7.54 (11.8) | 13.8 (12.1) |  | 12.5 (12.3) |
| Median [Min, Max] | 4.35 [0.300, 89.1] | 11.1 [0.310, 73.7] |  | 9.40 [0.300, 89.1] |
| **Total Hair Mercury (µg/g)** | |  | <0.001 |  |
| Mean (SD) | 1.92 (1.76) | 3.70 (3.20) |  | 3.34 (3.05) |
| Median [Min, Max] | 1.28 [0.167, 6.79] | 2.95 [0.00260, 21.4] |  | 2.61 [0.00260, 21.4] |

Fisher’s Exact Test for categorical and T-tests for continuous variables

Significance: <0.001‘***’; 0.001 ‘**’; 0.01 ‘*’; 0.05 ‘†’

**Supplemental Table 5:** Descriptive statistics of ACR and EATM studies to evaluate blood lead levels and anaemia. Fisher’s Exact Test for categorical and T-tests for continuous variables were used to evaluate differences between the ACR and EATM study.

|  | **EATM** | **ACR** | **p-value** | **Overall** |
| --- | --- | --- | --- | --- |
|  | **(N=48)** | **(N=201)** |  | **(N=249)** |
| **Age (Years)***** |  |  | <0.001 |  |
| Mean (SD) | 19.6 (13.9) | 36.1 (11.1) |  | 32.9 (13.4) |
| Median [Min, Max] | 20.5 [2.00, 49.0] | 35.0 [15.0, 66.0] |  | 33.0 [2.00, 66.0] |
| **Sex** |  |  |  |  |
| Female | 33 (68.8%) | 112 (55.7%) |  | 145 (58.2%) |
| Male | 15 (31.3%) | 89 (44.3%) |  | 104 (41.8%) |
| **BMI***** |  |  | <0.001 |  |
| Mean (SD) | 23.1 (7.56) | 28.1 (4.17) |  | 27.1 (5.36) |
| Median [Min, Max] | 22.1 [13.7, 39.9] | 27.9 [17.0, 42.6] |  | 27.3 [13.7, 42.6] |
| **Smoke Status**** |  |  | 0.002 |  |
| No | 46 (95.8%) | 150 (74.6%) |  | 196 (78.7%) |
| Yes | 2 (4.2%) | 51 (25.4%) |  | 53 (21.3%) |
| **Household Water Source** | |  |  |  |
| Treated | 31 (64.6%) | 120 (59.7%) |  | 151 (60.6%) |
| Untreated | 17 (35.4%) | 81 (40.3%) |  | 98 (39.4%) |
| **Cooking Fuel** |  |  |  |  |
| High Emissions | 20 (41.7%) | 62 (30.8%) |  | 82 (32.9%) |
| Low Emissions | 28 (58.3%) | 139 (69.2%) |  | 167 (67.1%) |
| **Education***** |  |  | <0.001 |  |
| Elementary | 28 (58.3%) | 28 (13.9%) |  | 56 (22.5%) |
| Middle School | 4 (8.3%) | 46 (22.9%) |  | 50 (20.1%) |
| High School | 14 (29.2%) | 103 (51.2%) |  | 117 (47.0%) |
| Advanced | 2 (4.2%) | 24 (11.9%) |  | 26 (10.4%) |
| **Highway Access***** |  |  | <0.001 |  |
| Highway | 34 (70.8%) | 23 (11.4%) |  | 57 (22.9%) |
| Non-Highway | 14 (29.2%) | 178 (88.6%) |  | 192 (77.1%) |
| **Road Access***** |  |  | <0.001 |  |
| Mean (SD) | 0.755 (0.421) | 0.507 (0.361) |  | 0.555 (0.385) |
| Median [Min, Max] | 1.00 [0, 1.00] | 0.750 [0, 1.00] |  | 0.750 [0, 1.00] |
| **Fish Consumption***** |  |  | <0.001 |  |
| Rarely | 0 (0%) | 24 (11.9%) |  | 24 (9.6%) |
| Monthly | 6 (12.5%) | 115 (57.2%) |  | 121 (48.6%) |
| Weekly | 42 (87.5%) | 62 (30.8%) |  | 104 (41.8%) |
| **Beef Consumption***** |  |  | <0.001 |  |
| Never | 17 (35.4%) | 19 (9.5%) |  | 36 (14.5%) |
| Monthly | 6 (12.5%) | 117 (58.2%) |  | 123 (49.4%) |
| Weekly | 25 (52.1%) | 65 (32.3%) |  | 90 (36.1%) |
| **Chicken Consumption***** | |  | <0.001 |  |
| Never | 1 (2.1%) | 2 (1.0%) |  | 3 (1.2%) |
| Monthly | 0 (0%) | 35 (17.4%) |  | 35 (14.1%) |
| Weekly | 31 (64.6%) | 164 (81.6%) |  | 195 (78.3%) |
| Daily | 16 (33.3%) | 0 (0%) |  | 16 (6.4%) |
| **Wild Game Consumption***** | |  | <0.001 |  |
| Never | 28 (58.3%) | 61 (30.3%) |  | 89 (35.7%) |
| Monthly | 12 (25.0%) | 114 (56.7%) |  | 126 (50.6%) |
| Weekly | 8 (16.7%) | 26 (12.9%) |  | 34 (13.7%) |
| **Native Status**** |  |  | 0.002 |  |
| Non-Native | 36 (75.0%) | 99 (49.3%) |  | 135 (54.2%) |
| Native | 12 (25.0%) | 102 (50.7%) |  | 114 (45.8%) |
| **Haemoglobin Level***** |  |  | <0.001 |  |
| Mean (SD) | 12.2 (1.39) | 13.6 (1.17) |  | 13.4 (1.34) |
| Median [Min, Max] | 12.1 [10.5, 15.1] | 13.5 [12.0, 17.7] |  | 13.2 [10.5, 17.7] |
| **Anaemia Status***** |  |  | <0.001 |  |
| Did Not Present Anaemia | 25 (52.1%) | 201 (100%) |  | 226 (90.8%) |
| Presented Anaemia | 23 (47.9%) | 0 (0%) |  | 23 (9.2%) |
| **Blood Lead Level (µg/dL) *** | |  | 0.027 |  |
| Mean (SD) | 2.72 (2.88) | 3.92 (3.49) |  | 3.69 (3.41) |
| Median [Min, Max] | 1.46 [0.250, 12.7] | 2.40 [0.520, 17.4] |  | 2.30 [0.250, 17.4] |
| **Blood Mercury Level (µg/L) **** | |  | 0.001 |  |
| Mean (SD) | 7.90 (9.09) | 14.7 (12.7) |  | 13.4 (12.4) |
| Median [Min, Max] | 4.00 [0.300, 47.0] | 12.4 [0.310, 73.7] |  | 11.1 [0.300, 73.7] |

Significance: <0.001‘***’; 0.001 ‘**’; 0.01 ‘*’; 0.05 ‘†’

**Supplemental Table 6:** Random mixed effect model results to test whether haemoglobin is associated with increased blood lead in the pooled data set (n = 249) with community as a random effect.

|  | **Univariate Models** | | **Multivariate Model** | |
| --- | --- | --- | --- | --- |
| **Risk Factor** | **Estimate** | **95% CI** | **Estimate** | **95% C.I.** |
| Sex (Ref: Female) | 0.17*** | 0.10 – 0.23 | 0.17*** | 0.11 – 0.24 |
| Age (Years) | 0.0008 | -0.002 – 0.003 | 0.0002 | -0.002 – 0.003 |
| Haemoglobin | -0.01 | -0.03 – 0.02 | -0.009 | -0.04 – 0.02 |

Significance: <0.001‘***’; 0.001 ‘**’; 0.01 ‘*’; 0.05 ‘†’

**Supplemental Table 7:** Descriptive statistics for the Aetiology of Anaemia and Trace Metals (EATM) study.

|  | EATM Sub-dataset |
| --- | --- |
|  | (N=123) |
| **Age (Years)** |  |
| Mean (SD) | 19.5 (13.8) |
| Median [Min, Max] | 21.0 [2.00, 69.0] |
| **Sex** |  |
| Female | 81 (65.9%) |
| Male | 42 (34.1%) |
| **Smoke Status** |  |
| No | 94 (76.4%) |
| Yes | 3 (2.4%) |
| Missing | 26 (21.1%) |
| **Community Type** |  |
| Mining | 62 (50.4%) |
| Native | 27 (22.0%) |
| Urban | 34 (27.6%) |
| **Household Water Source** |  |
| Treated | 86 (69.9%) |
| Untreated | 33 (26.8%) |
| Missing | 4 (3.3%) |
| **Cooking Fuel** |  |
| High Emissions | 46 (37.4%) |
| Low Emissions | 76 (61.8%) |
| Missing | 1 (0.8%) |
| **Education** |  |
| Advanced | 9 (7.3%) |
| Elementary | 61 (49.6%) |
| High school | 30 (24.4%) |
| Middle school | 19 (15.4%) |
| Unknown | 4 (3.3%) |
| **Beef Consumption** |  |
| Daily | 7 (5.7%) |
| Weekly | 54 (43.9%) |
| Monthly | 26 (21.1%) |
| Never | 34 (27.6%) |
| Missing | 2 (1.6%) |
| **Chicken Consumption** |  |
| Daily | 50 (40.7%) |
| Weekly | 63 (51.2%) |
| Monthly | 8 (6.5%) |
| Never | 2 (1.6%) |
| **Wild Game Consumption** | |
| Weekly | 16 (13.0%) |
| Monthly | 40 (32.5%) |
| Never | 67 (54.5%) |
| **Yuca Consumption** |  |
| Daily | 25 (20.3%) |
| Weekly | 65 (52.8%) |
| Monthly | 21 (17.1%) |
| Never | 12 (9.8%) |
| **Eat Yuca Daily** |  |
| No | 98 (79.7%) |
| Yes | 25 (20.3%) |
| **Haemoglobin** |  |
| Mean (SD) | 11.8 (1.25) |
| Median [Min, Max] | 11.3 [9.90, 15.1] |
| Missing | 11 (8.9%) |
| **Blood Lead Level (µg/dL)** |  |
| Mean (SD) | 2.70 (2.68) |
| Median [Min, Max] | 1.64 [0.250, 14.4] |
| **Blood Mercury Level (µg/L)** | |
| Mean (SD) | 7.06 (10.2) |
| Median [Min, Max] | 3.90 [0.300, 89.1] |
| **Total Hair Mercury (µg/g)** | |
| Mean (SD) | 1.84 (1.81) |
| Median [Min, Max] | 1.03 [0.128, 7.89] |
| Missing | 22 (17.9%) |

**Supplemental Table 8.** Random mixed effect model results for individuals who eat wild game (weekly or monthly) and log10 blood lead levels (µg/dL) with community as a random effect, using only the ACR dataset (n = 245).

| Risk Factor | Estimate | 95% Confidence Interval |
| --- | --- | --- |
| Sex (Ref: Female) | 0.13*** | 0.06-0.19 |
| Native (Ref: Non-indigenous) | 0.40*** | 0.28-0.53 |
| Eat Wild Game (Ref: Never) | 0.12** | 0.04-0.21 |

Significance: <0.001 ‘ *** ’; 0.001 ‘ ** ’; 0.01 ‘ * ’; 0.05 ‘ † ’
