# Supplementary material for: Risk of lead exposure from wild game consumption from cross-sectional studies in Madre de Dios, Peru
Source: Lancet Reg Health Am. 2022 May 8;12:100266. doi: 10.1016/j.lana.2022.100266 (PMC9555248; doi:10.1016/j.lana.2022.100266)
Supplement: Supplementary file 4 [file mmc4.docx]

***Editorial disclaimer:*** *This translation in Spanish was submitted by the authors and we reproduce it as supplied. It has not been peer reviewed. Our editorial processes have only been applied to the original abstract in English, which should serve as reference for this manuscript.*

**Abstracto**

Antecedentes: Estudios han mostrado niveles elevados con plomo en residentes de comunidades lejanas en la Amazonia; sin embargo, no todas las fuentes de exposición están completamente identificadas, como la exposición a plomo por el consumo de carne de monte cazada con balas de plomo.

Métodos: Los datos provienen de dos estudios que reclutaron a 307 individuos en 26 comunidades. Se utilizaron modelos de regresión con efectos aleatorios comunitarios para evaluar los factores de riesgo de LBL, incluida la dieta, la fuente de agua, el tabaquismo, el sexo, la edad y el estado indígena. El Modelo de Plomo para Todas las Edades (AALM) de la EPA se utilizó para estimar el fondo y la dosis del consumo de caza silvestre.

Resultados: El estado indígena y el consumo de caza silvestre se asociaron con un aumento de los niveles elevados de plomo en la sangre. Los participantes indígenas tuvieron 2,52 μg/dL (IC del 95%: 1,95 a 3,24) niveles más altos en comparación con los no indígenas. El consumo de caza silvestre se asoció con un aumento de 1,41 μg/dL (IC del 95%: 1,20 – 1,70) de plomo en la sangre. Consumir dos o más porciones de carne se asoció con un aumento de BLL de 1,66 μg/dL (IC del 95%: 1,10 – 2,57) en comparación con porciones más pequeñas. Usando el AALM, estimamos que las exposiciones al plomo son de 20 μg/día con el consumo de caza silvestre, lo que contribuye con 500μg/ comida. Por último, encontramos una fuerte asociación entre la exposición al plomo y al mercurio.

Interpretación: El consumo de carne de monte cazada con balas de plomo podría ser una fuente común de la exposición a plomo en la Amazonia. Comunidades que dependen de carne de monte y de la pesca podrían tener una exposición doble a plomo y mercurio, respectivamente.

Financiamiento Los fondos para realizar este estudio provinieron de las siguientes organizaciones: Duke Bass Connections, Duke Superfund Centre fundado por el National Institute of Environmental Health Sciences (P42ES010356), Duke Global Health Doctoral Scholar’s Program y Hunt Oil.
